# Effectiveness of pneumococcal conjugate 13-valent vaccine against severe pneumonia in Panama: a matched case-control study

**DOI:** 10.1016/j.jped.2025.03.008

**Published:** 2025-05-10

**Authors:** Jacqueline Levy, Rodrigo DeAntonio, Xavier Sáez-Llorens

**Affiliations:** a Hospital del Niño Doctor José Renán Esquivel, Provincia de Panamá, Panamá; b The Panama Clinic, Provincia de Panamá, Panamá; c Centro de Vacunación e Investigación (CEVAXIN) The Panama Clinic, Provincia de Panamá, Panamá; d Sistema Nacional de Investigación Panamá, Provincia de Panamá, Panamá

**Keywords:** Severe pneumonia, Children, Panama, Pneumococcal conjugate vaccine, Community-acquired pneumonia

## Abstract

**Objective:**

In Panama, the 13-valent pneumococcal conjugate vaccine (PCV13) was included in the primary immunization schedule in 2010 with a 3-dose schedule. The authors evaluated the effectiveness of PCV13 against severe community-acquired pneumonia in children of Panama after its introduction into the national immunization program.

**Methods:**

A retrospective matched case-control study was conducted at Hospital del Niño Doctor José Renán Esquivel, collecting data from children 2 to 59 months of age in years subsequent to the introduction of the PCV13 vaccine (2013–2015). Cases of severe community-acquired pneumonia had radiographically confirmed pneumonia (consolidated or with pleural effusion) or pneumonia with “other infiltrate” associated with CRP ≥ 40 mg/L with severity criteria according to the 2013 World Health Organization definition. Controls were children hospitalized for non-immune-preventable diseases matched by cases' age and admission date. Vaccine effectiveness was estimated as (1 - odds ratio) × 100 % with 95 % confidence intervals.

**Results:**

78 paired cases with 198 controls were included. In the cases, the mean age was 13.7 ± 10.3 SD months, and the hospital stay was 9.7 + 6.1 days. Overall, the effectiveness of PCV13 against severe community-acquired pneumonia was 54.0 % (95 % CI 25.0–72.0 %, *p* < 0.05). Vaccine effectiveness among children under 1 year was 61 % (95 % CI: 23.0–81.0 %) and 43 % (95 % CI:16.0–74.0 %) for children 1 to 4 years. For children who received at least 1 PCV13 dose was 17.2 % (95 % CI: 8.8–33.7 %). Overcrowding and lack of vaccination against influenza were risk factors for lower vaccine effectiveness.

**Conclusions:**

PCV13 was effective in preventing severe cases of community-acquired pneumonia in children in Panama.

## Introduction

Streptococcus pneumoniae (*S. pneumoniae* or pneumococcus) is considered the leading cause of bacterial community-acquired pneumonia (CAP) worldwide and the second leading cause of pneumonia hospitalizations after respiratory syncytial virus. CAP is the leading cause of death among children aged 1 to 59 months worldwide.^1^ Among the causes of death from pneumococcal infections, pneumonia accounts for 81 % in low and middle-income countries,^2^ and the most susceptible populations are children under 5 years of age, with 0.8 million deaths in 2018.^3^ and population over 65 years. In Latin America and the Caribbean (LAC), pneumonia is responsible for 14 % of deaths in children under 5 years.^4^

A systematic analysis reported that lower respiratory infections caused 13.1 % of all deaths worldwide in children under 5 years of age. S. pneumoniae was the leading cause of morbidity and mortality from lower respiratory infections globally, contributing to more deaths than all other etiologies combined in 2016.^5^

The pneumococcal conjugate vaccine (PCV) has been recommended by the World Health Organization (WHO) to prevent CAP.^5^ WHO recommends its inclusion in the Expanded Program on Immunization (*EPI*) of countries with high CAP-related morbidity and mortality.^6^^,^^7^ After introducing PCVs worldwide, the burden of pneumococcal diseases significantly decreased in children. It is recognized that introducing these vaccines has been a good strategy for immunization in the population at risk and eliminating nasopharyngeal transmission, in addition to indirectly contributing to the reduction of antimicrobial resistance.^6^ However, since 2015, several LAC countries have reported an increased incidence of pneumococcal disease in non-vaccine serotypes,^6^^,^^7^^,^^8^ which led to questions regarding the long-term benefit of PCVs.

In 2008, the 7-valent pneumococcal conjugate vaccine (PCV7) was introduced into the *EPI* of the Republic of Panama; later, at the end of 2010, it was changed to PCV13. Since its introduction, it has been placed in a 2 + 1 scheme (2 months, 4 months, and 1 year of age), and it is estimated that the coverage of this vaccine at the national level is currently 90 %.^8^

Several studies of the impact and effectiveness of PCVs have been conducted after their implementation in LAC, in addition to epidemiological surveillance with SIREVA II (Surveillance Network System of Agents Responsible for Bacterial Pneumonia and Meningitis),^9^ which provides prospective information on the distribution data of serotypes and susceptibility of S. pneumoniae to antibiotics, as well as epidemiological information for estimating the burden of these diseases and the formulation of increasingly efficient vaccines. However, PCVs' effectiveness has not been studied in the Panamanian population since its introduction, which is very important to generate real-life evidence for the sustainability of *EPI* programs.^10^

In recent years, pneumonia has been mainly severe, with an average of 1668 hospitalizations per year.^11^ Respiratory diseases are among the five leading causes of morbidity in patients hospitalized at the Hospital del Niño doctor José Renán Esquivel, accounting for approximately 30 % overall, according to the hospital epidemiological bulletin.^12^ Mortality is lower, compared to other diseases reported in the epidemiological bulletin as neonatal conditions and other infectious diseases, but still representing 5 % of the causes of death in patients.^11^ Most of the pneumonia cases at the hospital corresponded to severe as per WHO definition.^13^

This study, conducted at one of the largest reference pediatric hospitals in the Republic of Panama, represents an opportunity to evaluate the effectiveness of pneumococcal vaccination in children under 5 years of age in real life where the largest number of cases of pneumonia from different regions of the country are admitted.

## Materials and methods

A matched case-control study was conducted identifying severe pneumonia cases during the 2013–2015 period. The case definition corresponded to all children between 2 and 59 months hospitalized for severe CAP according to the WHO severity criteria. Cases without information about vaccination status or available radiological images were excluded. For each case, between one and three controls were included, matched by age and date of admission. The controls were children between 2 and 59 months hospitalized with a discharge diagnosis unrelated to vaccine-preventable diseases. Cases and controls were matched based on age group, geographic location, hospitalization date, and presence of comorbidities Children with human immunodeficiency virus (HIV) and sickle cell disease were excluded as controls. To optimize statistical efficiency while considering feasibility constraints based on the difficulties identifying cases, the authors evaluated different case-to-control ratios (1:1, 1:2, and 1:3). Based on standard epidemiological guidelines and sample size calculations, a ratio of 1:2 or 1:3 was considered as deemed appropriate to enhance power without unnecessarily increasing resource challenges. The estimated sample size required for this case-control study to assess a vaccine effectiveness of 50 %, with a 50 % vaccine coverage among controls, 95 % confidence level, and 80 % power was:

• For a 1:2 case-to-control ratio → 45 cases and 90 controls.
• For a 1:3 case-to-control ratio → 40 cases and 120 controls.

The sample size was achieved based on the estimation described above.

According to the WHO, pneumonia was defined as any child from 2 to 59 months admitted to the hospital with fever, shortness of breath, and tachypnea at rest according to age (≥50 breaths/minute 2 to 11 months - ≥40 breaths/minute 12 to 59 months). To define severe CAP, radiological images were assessed by a certified radiologist. Some other causes for severe pneumonia in Panamanian children are associated with viral (RSV and influenza) and bacterial pathogens (*Mycloplasma pneumoniae, Bordetella pertussis* and *Haemophilus influenzae*). Children were included after a review of clinical records was undertaken focused on identifying the presence or absence of specific signs and symptoms that are indicative of severity criteria. Vaccination information was obtained from the cases and controls vaccination card or from medical chart information. Subsequently, data was collected using a data collection sheet. Children were classified as having severe pneumonia in the presence of at least one of the following: (a) severe respiratory distress or (b) signs of pneumonia with a general alert sign (inability to drink or breastfeed, daily vomiting, seizures, lethargy or reduced level of consciousness, thoracic retractions, stridor at rest, apnea).

The sample size was calculated considering 3 elements: the expected effectiveness of vaccination for bacterial CAP, the number of controls for each case, and the expected vaccination coverage in the controls (≈50 %).

Information was collected on the following variables: Influenza vaccination, weight, maternal education, low family income (monthly household income per member less than or equal to 50 % of the standard monthly minimum wage), overcrowding (more than 3 people sleeping with the child in the same room).

### Data analysis

The data was analyzed using the STATA 14.0 software. A descriptive analysis was performed to determine the characteristics of the cases and controls. Mean and standard deviation were used for quantitative variables, and proportions for categorical variables. Comparisons between the characteristics of cases and controls were made using *t*-tests and Chi-square tests.

In this matched case-control study, vaccine effectiveness (VE) was estimated using the odds ratio (OR), which represents the odds of vaccination among cases compared to controls. The formula for VE used was: VE = (1−OR) × 100 where OR was calculated using a conditional logistic regression model, accounting for the matched pairs of cases and controls and adjusting for potential confounders such as age, comorbidities, and healthcare access. The VE was expressed as a percentage, representing the reduction in disease risk among vaccinated individuals compared to unvaccinated individuals.^14^ The primary analysis included all children who had received a complete PCV series compared to children with an incomplete series for age.

A secondary analysis was performed on all children who received a full or partial series of PCV compared to children who had not received vaccine doses. The analyses were performed using conditional logistic regression to adjust for different variables.

The study evaluated risk factors, confounding factors, possible interaction, and collinearity as part of the multivariable conditional logistic regression modeling process. The final models considered associations with *p* values < 0.05 as statistically significant.

**Figure 1:**
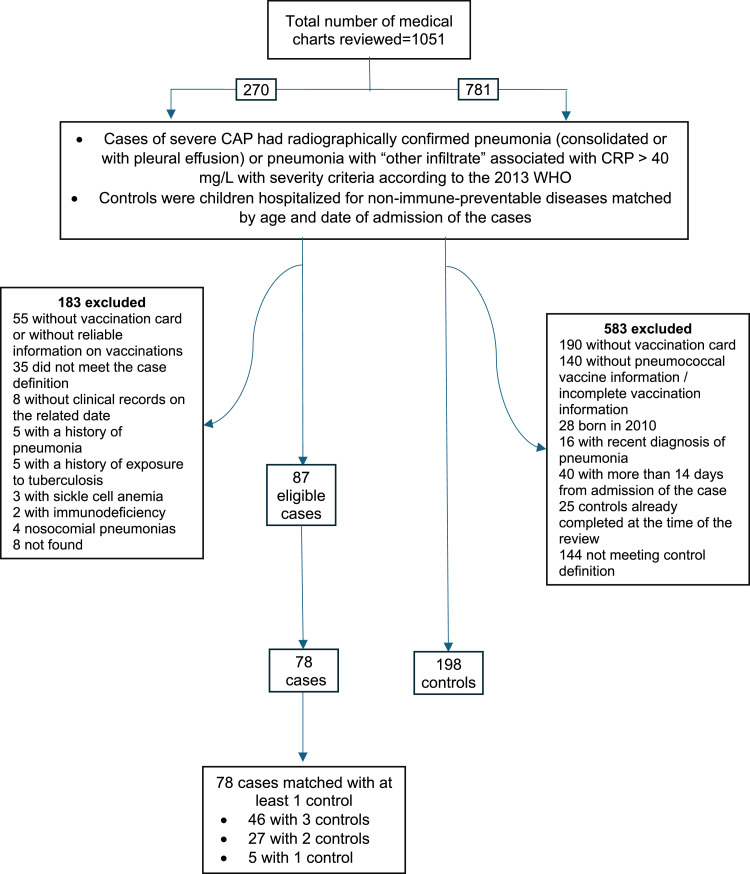
Study flow diagram.

All reasons for the exclusion of cases and controls are described in Figure 1.

### Ethical approval

Access to children's information was made by responsible healthcare personnel and in accordance with national standards. All personal identification data were removed from the analysis databases. The study was approved by the Institutional BioEthics Committee of the Hospital del Niño Dr. Jose Renan Esquivel in Panama.

## Results

Overall, 78 cases paired with 198 controls were included during the 2013–2015 period Figure 1.

### Cases

Of 78 severe pneumonia paired cases, 53 % of the children were male, and 47 % were female. The majority of the cases were from Central Panama (52.5 %), followed by Western Panama (12.8 %) and the Guna-Yala region (9.0 %). According to the study radiologist's description of the chest X-ray, consolidation was present in 43.5 % of the cases, the presence of infiltrates in 42.3 %, and pleural effusion in 14.1 %. Additionally, 71.9 % of children required admission to the intensive care unit, and 66.7 % required mechanical ventilation. Regarding the laboratory parameters, the leukocytes were elevated with a mean of 15,431 (SD ± 8340) 10^∧^3/uL, and the C-reactive protein was ≥ 40 mg/L in 91 % of the cases.

It was observed that 25.6 % of the children completed the three-dose vaccination with PCV13 as part of the routine childhood immunization, 32.1 % of the children received two doses of the vaccine, 25.6 % of the children received one dose, and 16.7 %, did not receive any doses of the vaccine.

### Controls

Of 198 controls, 55.6 % of the children were male, and 44.4 % were female. The majority of the cases were from Central Panama (50 %), followed by Western Panama (21.2 %) and Colon (9.6 %). It was observed that 36.9 % of the children completed the three-dose vaccination as part of the routine childhood immunization, 45.4 % of the children received two doses of the vaccine, 14.1 % of the children received one dose, and 3.6 %, did not receive any doses of the vaccine.

Demographic characteristics of children are presented in Table 1.

**Table 1:** Study population characteristics and risk factors

Variable	Cases (*N* = 78) n(%)	Controls (*N* = 198) n(%)	*p* value
Age (months) media; ± SD	13.3 ± 10.3	13.9 ± 10.3	0.64
Chronic diseases history	10 (12.8)	40 (20.2)	0.28
Low Weight	20 (25.6)	33 (16.7)	0.03
No education	11 (14.1)	3 (1.6)	<0.01
Low income	31 (39.7)	89 (45.0)	0.26
Overcrowded	40 (51.2)	34 (17.2)	0.000
Breastfeeding history	70 (89.9)	168 (84.9)	0.465
Influenza vaccination	18 (23.0)	89 (45.0)	0.001
Hospital stay (days) media; ± SD	9.7 + 6.1	6.5 + 5.4	<0.01
Number of PCV doses			
0	13 (16.7)	7 (3.6)	
1	20 (25.6)	28 (14.1)	0.048
2	25 (32.1)	90 (45.4)	0.001
3	20 (25.6)	73 (36.9)	0.001

Definitions: **Low weight,** Growth in height and weight of a person. It is measured using the body mass index (BMI) in people over 2 years of age and Weight/age in people under 2 years of age; **No education,** Level of schooling completed by the mother; **Low income,** Monthly household income per member less than or equal to 50 % of the standard monthly minimum wage; **Overcrowded,** It represents the quotient between the total number of people in the home and the total number of rooms or rooms available in the home; **Breastfeeding history,** Having received breastmilk at any time.

SD, Standard deviation; PCV, Pneumococcal conjugate vaccine.

VE = (1−OR) × 100.

### Vaccine effectiveness

**Figure 2:**
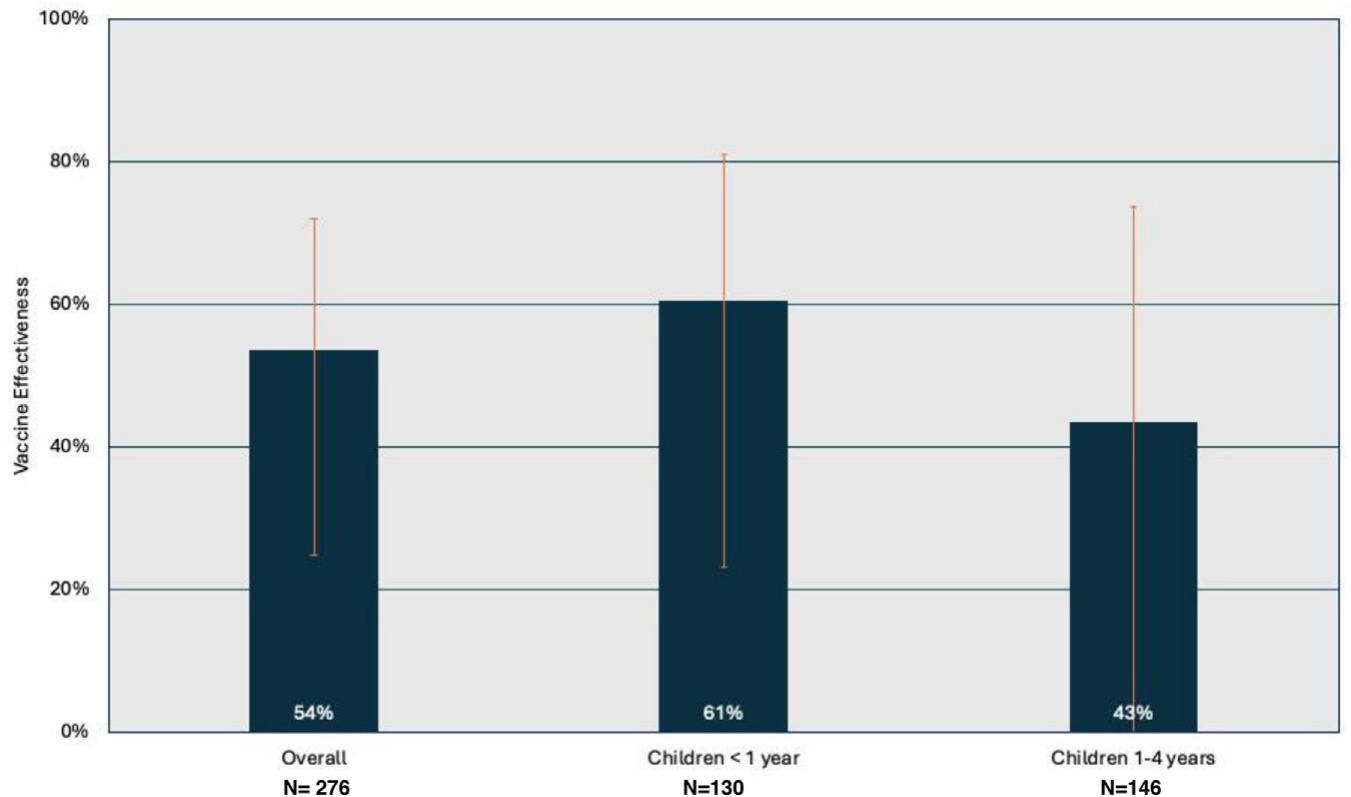
PCV13 Vaccine effectiveness by age group.

The VE for severe pneumonia was 54.0 % (95 % CI 25.0–72.0), but when evaluating VE according to complete and incomplete vaccination schemes by age group, the VE for children under 1 year of age was 61 % (95 % CI 23.0–72.1) and for children 1 – 4 years was 43 % (95 % CI −16.0–73.8 %) Figure. 2.

The variables identified as factors that negatively influence the performance of the vaccine with an OR > 1, were living in overcrowded, being underweight, having no maternal education, and lack of influenza vaccination. Age (mainly infants) represented a risk factor for pneumonia cases that had not received vaccination.

Vaccination with 2 or 3 doses of the PCV13 vaccine showed effectiveness. However, a statistically significant positive effect was also observed with 1 dose. Variables such as chronic disease, breastfeeding, and low family income did not have statistical significance. An adjusted multivariate logistic regression analysis is presented in Table 2.

**Table 2:** Adjusted Multivariate logistic regression analysis – matched case-control

	Odds Ratio	CI 95 %		*p*-value	
**Age**					
0–5 months	Reference				
6–11 months	2.91	1.32	6.41	0.008	*
12–23 months	3.84	1.57	9.42	0.003	*
24–59 months	3.97	1.41	11.16	0.009	*
**Sex**					
Female	Reference				
Male	0.84	0.53	1.33	0.457	
**Chronic disease**					
Yes	0.91	0.48	1.72	0.774	
**Overcrowded**					
Yes	3.33	1.95	5.68	<0.0001	*
**Previous breastfeeding**					
	0.58	0.25	1.35	0.465	
**Income**					
Low income	0.71	0.39	1.29	0.26	
**Weight**					
Normal	Reference				
Low weight	1.91	1.08	3.40	0.03	
Overweight	0.54	0.24	1.19	0.13	
Obesity	0.28	0.07	1.09	0.07	
**Mother education level**					
University	Reference				
None	2.74	0.70	10.72	0.15	
Elementary	1.30	0.58	2.93	0.52	
High school	1.06	0.51	2.18	0.88	
**Influenza vaccination**					
No	2.59	1.44	4.66	0.001	*
**No. of PCV doses**					
None	Reference				
1	0.34	0.12	0.99	0.048	*
2	0.17	0.06	0.47	0.001	*
3	0.14	0.05	0.45	0.001	*

*CI*, Confidence interval; PCV, Pneumococcal conjugate vaccine.

## Discussion

This case-control study is the first study in Panama to demonstrate the effectiveness of the 13-valent pneumococcal conjugate vaccine (PCV13) in preventing hospitalizations for severe bacterial CAP. The study was conducted in a real-world setting once the vaccine was implemented as part of routine childhood immunization.

Vaccine effectiveness was 54 % for severe pneumonia. These data can be compared with those found in a study conducted in Rwanda, where the VE of PCV13 for severe pneumonia was found to be 54 %,^15^ unlike a study in India where the effectiveness was 31 %.^16^ This resemblance to a systematic review of 2023, reinforced the current global evidence of the effectiveness of PCV13 against pneumonia,^17^ suggesting the role that PCV13 has in reducing severe pneumonia hospitalizations. Similarly, a systematic review in LAC that compiled the impact and effectiveness of PCV studies (Brazil, Chile, Uruguay, Argentina, Peru, and Nicaragua) from 2009 to 2016 reported that effectiveness ranged from 8.8 to 37.8 % for hospitalizations due to X-ray confirmed pneumonia and showed markedly high PCV13 VE estimates and impact of PCVs on hospitalization due to pneumonia in children less than 5 years old.^18^

The variables with statistical significance identified as factors that negatively impact the vaccine's performance were living in overcrowded, lack of education of the parent/guardian, and lack of influenza vaccination. Suffering from a chronic condition generally represents a negative factor for the performance of the vaccine; however, in this study, the percentage of comorbidities was not high and therefore did not have statistical significance, unlike most studies in which it shows to be a significant factor, emphasizing diseases such as asthma and congenital heart disease.^19^

The introduction of pneumococcal conjugate vaccines in the *EPI* in developed and developing countries has impacted the reduction of the incidence of pneumococcal disease and has demonstrated its effectiveness, with percentages that vary depending on the area and population studied.

### Limitations

The study had several limitations, typical of a case-control study.^20^^,^^21^ As this is a retrospective study, the authors depend on the existing information, and it is impossible to collect additional valuable information to obtain better results and the desired sample size. In this particular study, it occurred that initially not all chest x-rays were available for evaluation and later when reviewing the records, many did not have the vaccine report. Specifically, the information on the pneumococcus vaccine was not clean, so several patients who met the case definition could not be included. Another limitation of the study is that there was no serotype information, and therefore, VE against vaccine serotypes could not be determined. Also, no information about viral co-infections was available.

PCV13 was effective in preventing severe cases of CAP in children of Panama. The evaluation of vaccine effects in the individual and the population in real-life settings helps to understand the complex dynamics of pneumonia epidemiology following changes in the pneumococcal vaccination program.

In Panama, the 13-valent pneumococcal vaccine included in the national immunization schedule at the end of 2010 proved to be highly effective in preventing severe pneumonia when completing the 3-dose schedule. However, as is historically known, serotype replacement would need the development of new vaccines. Considering this, the authors must continue strengthening the surveillance systems to identify potential changes in disease trends.

A limitation of the study was the lack of pneumococcus-positive culture data and pneumococcal serotype data as many vaccine effectiveness studies focus more on the vaccine effectiveness against pneumococcal infections. Nevertheless, the authors demonstrated effectiveness even for children under 1 year of age.

## Source of funding

The research was funded by the authors.

## Authors contributions

Authors contributions: JL, XSL, and RD conceptualized the study design and analyzed the data. JL collected the data and supervised the conduction of the study. All authors were significant contributors in writing the manuscript. All authors read and approved the final manuscript.

## Data access

Available upon request.

## Conflicts of interest

The authors declare no conflicts of interest.
